# Functioning pancreatic neuroendocrine neoplasms: diagnosis and current treatment

**DOI:** 10.1530/EO-26-0005

**Published:** 2026-10-09

**Authors:** Panagiota Konstantakou, Ariadni Spyroglou, Marina Tsoli, Anna Angelousi, Gregory Kaltsas, Krystallenia I Alexandraki

**Affiliations:** ^1^ Second Department of Surgery, Medical School, Aretaieio Athens Hospital, National and Kapodistrian University of Athens, Athens, Greece; ^2^ Neuroendocrine Tumour Unit, ENETS Centre of Excellence & EURACAN 4-Member, 1st Department of Propaedeutic Internal Medicine, Laiko Hospital, National and Kapodistrian University of Athens, Athens, Greece; ^3^ First Department of Internal Medicine, European Reference Network on Rare Endocrine Conditions (ENDO-ERN), Laikon General Hospital, Medical School, National and Kapodistrian University of Athens, Athens, Greece

**Keywords:** somatostatin analogs, rare functional pancreatic syndromes, insulinoma, gastrinoma, PRRT

## Abstract

Pancreatic neuroendocrine neoplasms (PanNENs) are rare and characterized by variable and heterogeneous clinical manifestations, comprising both symptoms and signs of tumor growth and systemic manifestations of hormonal hypersecretion. The diagnosis is challenging due to their rarity, neoplasm localization, and diverse secretory profiles. A subset of these neoplasms may develop in the context of inherited syndromes and harbor specific genetic alterations requiring genetic counseling of other family members. These particular features require a multidisciplinary approach for managing patients with a functioning PanNEN syndrome. Surgical resection of local disease, antisecretory medical treatment, locoregional treatment, and systemic treatments are employed on an individualized basis. The treatment landscape of advanced NENs progressing after systemic treatment or after exhibiting intolerance to sequential lines of treatment is rapidly evolving supporting the introduction of additional therapies with promising results.

## Introduction

Pancreatic neuroendocrine neoplasms (PanNENs) constitute a rare and heterogeneous group of slow-growing but potentially metastatic neoplasms with an increasing incidence over the last years (1). PanNENs are, in their majority, well-differentiated neuroendocrine tumors, classified into non-functioning and functioning neoplasms depending on the presence of a clinical syndrome combined with biochemical evidence of inappropriately elevated hormonal levels (2). Functioning PanNENs mainly include insulinomas and gastrinomas, followed by glucagonomas, vasoactive intestinal polypeptide (VIP)omas, somatostatinomas, PanNENs associated with carcinoid syndrome (CaS), parathyroid hormone-related protein (PTHrP)omas, growth hormone-releasing hormone (GHRH)omas, adrenocorticotropic hormone (ACTH)omas, and calcitoninomas (2). These tumors may either be sporadic or arise in the context of a hereditary syndrome (3), and their clinical and biochemical suspicion is followed by the localization of the neoplasm and staging to establish the diagnosis (2). Morphological imaging with computed tomography (CT) or magnetic resonance imaging (MRI), along with functional somatostatin receptor (SSTR) imaging (SRI) and fluorodeoxyglucose (FDG)-PET/CT in high-grade PanNEN, is required for localization (2, 4). Endoscopic ultrasound (EUS) can be helpful for the detection of small PanNENs (particularly small-sized insulinomas) and duodenal NENs (particularly gastrinomas embedded in the duodenal wall), as well as for the definition of the tumor location in relation to the pancreatic duct, particularly when minimally invasive enucleation is an option (2, 4, 5). Surgery with curative intent is the cornerstone of treatment for locoregional or locally advanced functioning PanNENs. In the presence of advanced metastatic disease, surgical locoregional therapies may play a role in decreasing disease burden and achieving symptom control (6, 7). Furthermore, if supportive treatment or surgical management is insufficient to control the functional syndrome, first-line therapy to reduce hormonal activity in SSTR-positive neoplasms is the somatostatin analogs (SSAs) (2, 8, 9). Systemic treatments with SSA or peptide receptor radionuclide therapy (PRRT) and chemotherapy (temozolomide/capecitabine (Tem/Cap) or streptozotocin (SZT)-based therapies) or molecular targeted treatments (MTT) play a role in personalized management for the control of both tumor growth and hormonal syndrome (9, 10).

In this review, we will present the clinical spectrum of functioning PanNENs and the diagnostic considerations and related algorithms and discuss the treatment options depending on disease extent. The importance of the multidisciplinary team (MDT) approach that includes all relevant specialties will be highlighted, given the complexity and multisystemic involvement of these neoplasms.

## Clinical presentation and diagnostic considerations of functioning PanNENs

### Insulinoma

#### History and clinical presentation

Insulinoma is the most frequent functioning PanNEN with an incidence of 3–4 cases/million/year (2, 9, 11). Approximately 90% of insulinomas are sporadic and rarely malignant (5–10%) (2, 11). History of hypoglycemia in a nondiabetic patient along with the presence of Whipple’s triad (symptoms of hypoglycemia, documented low-plasma glucose, and resolution of symptoms after ingestion of carbohydrates) raises the suspicion of an underlying insulinoma (2). In cases of chronic hypoglycemia, autonomic symptoms are blunted or absent, and hypoglycemia manifests with neuroglycopenic symptoms (such as irritability, cognitive deficits, transient focal neurological deficits, seizures, and coma); however, these latter manifestations are less common in insulinoma patients, where rather mixed symptoms are present (2, 11, 12). The lack of specificity of hypoglycemic symptoms may delay biochemical evaluation and diagnosis by 2–5 years and occasionally longer (2, 11). Key points of symptoms’ history pointing to insulinoma diagnosis are the timing of symptoms (during fasting, particularly during the night) and significant weight gain (50% of patients diagnosed with insulinoma).

#### Biochemical assessment

If the suspicion of hypoglycemia has been raised, blood glucose monitoring is advised at the time of symptoms. In case of a patient with the aforementioned symptoms and documented spontaneous hypoglycemia (<45 mg/dL) in a plasma sample, insulin, C-peptide, and, if possible, proinsulin and β-hydroxy-butyrate should be measured simultaneously (2, 11). Often spontaneous hypoglycemia is not evident during outpatient observation, and the 72-h fasting test is performed in an inpatient setting. The diagnostic criteria for insulinoma during a 72-h fast are as follows: i) plasma glucose < 45 mg/dL, ii) plasma insulin > 3 μIU/mL, iii) plasma C-peptide > 200 pmol/L, iv) plasma proinsulin > 5 pmol/L, and v) plasma β-hydroxybutyrate < 2.7 nmol/L. About 42.5% of patients with insulinoma are symptomatic within the first 12 h, 67% by 24 h, and 95% by 48 h (13). On the other hand, approximately 5% of patients with insulinomas present post-prandial but not fasting hypoglycemia. In this latter case, a mixed meal test is the alternative option (2).

#### Imaging

Multiphasic contrast-enhanced CT imaging is required to localize these typically small hypervascularized lesions (14). The sensitivity of CT to detect small functioning tumors is as high as 83–88% during the arterial phase and lower up to 11–76% during the portal venous phase (15, 16). Insulinomas are usually depicted as solitary, rounded, homogeneously hyper-enhanced lesions in the arterial phase of CT (sensitivity 94%) without causing stenosis of the main pancreatic duct (14). EUS shows a high sensitivity of roughly 87%, a specificity of 98%, and allows fine-needle aspiration (FNA) biopsy/cytology, also enabling immunohistochemical staining of hormones of suspected lesions (17). EUS may detect lesions as small as 2–5 mm, whereas its additional advantage is the estimation of the distance of the neoplasm from the main pancreatic duct, a crucial piece of information when enucleation is considered (2). The overall diagnostic sensitivity for combined CT and EUS has been reported to be 100% (18). On MRI scanning, PanNENs are usually detected on diffusion-weighted imaging, hypointense on T1 and moderately hyperintense on T2 (19). The reported sensitivity and specificity of MRI for detecting insulinomas is 54 and 65%, respectively (20). Occasionally, the localization of insulinomas can be challenging with the traditional morphological imaging modalities. Molecular functional imaging provides the opportunity for noninvasive localization and characterization of insulinoma. Peptide receptors expressed in insulinomas such as SSTRs can be targeted by SRI, the glucagon-like peptide-1 receptor (GLP-1R) by ^68^Gallium (Ga)-exendin-4, and the amine precursor uptake and decarboxylation (APUD) system by 6-[^18^F]-l-fluoro-l-3,dihydroxyphenylalanine (^18^F-DOPA) (21, 22, 23, 24). ^68^Ga-exendin-4 showed a pooled sensitivity and specificity of 94 and 83%, respectively, or a sensitivity of 97.7%, in localized insulinomas, given the high GLP-1R expression of these neoplasms (25) outperforming other radiopharmaceuticals (22). However, when not available, ^68^Ga-radiolabeled somatostatin analog (^68^Ga-DOTA-SSA) can be used (21). This later radionuclide imaging is used with three compounds, namely [^68^Ga-DOTA0-Ty3]octreotate (DOTATATE), [^68^Ga-DOTA0Ty3]octreotide (DOTATOC), and [^68^Ga-DOTA0-NaI3]octreotide (DOTANOC), having varying SSTR subtype specificity with highest affinity for SSTR2, SSTR5, and SSTR3/5, respectively (26). However, insulinoma detection with ^68^Ga-DOTA-SSA is suboptimal, since these tumors have a limited SSTR2 expression profile, a small size, and a physiologically high ^68^Ga-DOTA-SSA uptake in the uncinate process (27). According to recent evidence, the overall sensitivity, specificity, and detection rate for DOTATOC PET/CT were 58, 89, and 64%, respectively, for the identification of insulinomas (23).

#### Arterial calcium stimulation with hepatic venous sampling (ASVS)

In the rare cases of occult functional insulinomas with negative or equivocal imaging, arterial calcium stimulation with hepatic venous sampling (ASVS) is the gold standard invasive procedure to localize the neoplasms. This procedure is performed after overnight fasting and includes an angiography combined with injections of calcium gluconate into the gastroduodenal, splenic, and superior mesenteric arteries. Calcium stimulates insulin release from insulinomas but not from normal islets. Insulin is then measured in the hepatic vein effluent, and a rise in insulin levels at 30 or 60 s (twofold or higher) in one or more of the catheterized arteries (in case of multiple tumors or a single insulinoma fed by two arteries) localizes the source of hyperinsulinism (28). A meta-analysis of 10 studies including 337 patients showed an ASVS sensitivity and specificity of 93 and 86%, respectively (29). Differential diagnosis of insulinoma includes non-insulinoma pancreatogenous hypoglycemia syndrome (NIPHS), an adult-onset form of nesidioblastosis frequently occurring post-bariatric surgery, and non-islet cell tumor hypoglycemia (NICTH) due to IGF2 hypersecretion by PanNENs (30, 31).

### Gastrinoma

#### History and clinical presentation

Gastrinoma is the second most frequent functioning PanNEN, with an incidence of 1–1.5 cases/million/year (32). The majority of gastrinomas are extra-pancreatic (pancreatic 25%), mostly duodenal (70%), with a percentage of 5–10% of non-duodenal pancreatic localization, while MEN1 gastrinomas, accounting for 20% of the cases, are mostly pancreatic (33). Gastrinomas exhibit aggressive features with malignant potential in nearly 60% of cases, mainly metastasizing to regional lymph nodes (33). Zollinger–Ellison syndrome (ZES) is associated with gastrin hypersecretion, inducing marked gastric acid hypersecretion either in sporadic cases or in the context of multiple endocrine neoplasia 1 (MEN1) (34). The diagnosis of gastrinoma should be suspected in the presence of peptic ulcer disease (PUD), severe gastroesophageal reflux disease (GERD), and/or diarrhea that responds to proton pump inhibitors (PPIs) (35). Furthermore, the presence of multiple ulcers of atypical localization, refractory to conventional treatment, in the absence of NSAID use or of an underlying disease, may raise the suspicion of gastrinoma (2).

#### Biochemical assessment

Fasting serum gastrin (FSG) levels >1,000 pg/mL (10-fold elevated) on two separate occasions in combination with the presence of a gastric fluid pH ≤ 2 when basal gastric acid output is over 15 mEq/h (or over 0.5 mEq/h in patients with previous vagotomy), or a ratio of basal to maximal acid output is over 0.6 are diagnostic of ZES (36). In patients with intermediate gastrin levels (150–1,000 pg/mL) and acid secretion, the provocative secretin stimulation test can confirm the diagnosis. An intravenous (IV) bolus of secretin 0.4 μg/kg is given with serial measurements of serum gastrin at baseline and 2, 5, 10, 15, 20, and 30 min after injection, and a gastrin increase >120 pg/mL from basal values is considered diagnostic of gastrinoma (sensitivity 94%, specificity 100%) (34). Discontinuation of PPIs for at least 7 days and substitution with high-dose H2-receptor antagonists until 12–24 h prior to any test is recommended. However, the secretin stimulation test and the basal gastric acid output are not any more widely available tests in everyday clinical practice. Evaluation of gastric fluid pH can be obtained through a nasogastric tube or esophagogastroduodenoscopy (OGD) using an electrode, a filter paper, or a biochemical evaluation of the aspirate. However, hypergastrinemia may be encountered also in alternative clinical conditions. Achlorhydria and hypochlorhydria states such as atrophic gastritis, *Helicobacter pylori* infection, renal disease, and chronic PPI use should be considered and excluded along with rarer causes of increased FSG (gastric outlet obstruction, short bowel syndrome, retained gastric antrum syndrome, and juvenile gastric polyposis) (2). A 5- to 7-year delay in diagnosis due to the confounding effect of PPI use has been reported (36). The risk of rebound gastric acid hypersecretion and consequent severe exacerbation up to perforation or bleeding must not be overlooked when attempting a PPI withdrawal in a subset of patients (2, 36).

#### OGD and imaging

To overcome the above-mentioned difficulties of the classic diagnostic biochemical assessment, complementary diagnostics are applied in contemporary clinical practice in expert centers. OGD, EUS, CT/MRI, and SRI are recommended (24). In patients with suggestive clinical symptoms and elevated FSG, the documentation of prominent gastric folds in OGD with a gastric pH ≤ 2 is strongly supportive of the diagnosis, when alternative diagnoses and secondary hypergastrinemia are ruled out (2, 37, 38).

## Rare functioning PanNENs

### Glucagonoma

#### History and clinical presentation

Glucagonoma is rare with an annual incidence of 0.01–0.1 new cases per 1,000,000 (11). The neoplasm is usually solitary and located in the distal pancreas. Patients typically present in their fifth decade (39). Most glucagonomas are sporadic, whereas up to 20% may be associated with MEN1. Glucagonomas are usually large (over 3 cm), and approximately 50–80% are already metastatic at diagnosis (11). Glucagonoma syndrome is defined by classic symptoms and signs such as weight loss (60% of patients), necrolytic migratory erythema (80%), and mucosal abnormalities, including stomatitis, cheilitis, and glossitis (40%), diabetes (70%), deep vein thrombosis (50%), and diarrhea (60%) along with glucagon hypersecretion (40, 41). Weight loss is secondary to the catabolic action of glucagon. Necrolytic migratory erythema is thought to develop secondary to a combination of poor nutrition, low zinc, and low amino acid levels and extends mainly to the perioral and perigenital regions, fingers, legs, and feet (41). Diabetes results from the direct metabolic effects of glucagon; diarrhea results from the increased glucagon levels and co-secretion of gastrin, VIP, serotonin, and calcitonin (42, 43). In rare cases, glucagonomas are associated with dilated cardiomyopathy that can be reversible after tumor control (44). Previously, the acronym 4D syndrome was used to denote dermatitis, diabetes, deep vein thrombosis, and depression. A rare and recently described autosomal recessive syndrome associated with hyperglucagonemia is glucagon cell hyperplasia and neoplasia of the endocrine pancreas, defined as Mahvash disease (45). Glucagon cell hyperplasia and neoplasia is a genetically determined receptor disease, affecting the glucagon receptor, inactivation of which interrupts glucagon signaling in the liver, with subsequent disturbed metabolism of glycogen, fatty acids and amino acids along with hyperplastic changes of the glucagon cells of the pancreatic islets and hyperglucagonemia (46).

#### Diagnosis

The combination of clinical features along with a fasting plasma glucagon >500 pg/mL and particularly >1,000 pg/mL supports the diagnosis of glucagonoma (2). CT and MRI of the abdomen are the morphological imaging studies for tumor localization. Since glucagonomas express abundant SSTRs, SRI use is mandatory (24, 47).

### VIPoma

#### History and clinical presentation

VIPomas are NENs with an estimated incidence of 0.1 new cases per 1,000,000 and can occur in both adults and children (48). In adults, they commonly appear at a mean age of 55 years and are mostly intrapancreatic (95%) (49). VIPomas are sporadic in 90% of cases; in about 5% of patients, they present in the context of MEN1 (50). Pancreatic VIPomas are generally solitary lesions > 3 cm, typically located in the body and tail of the pancreas, while 25% are in the head of the pancreas (51). The majority of VIPomas are malignant and have already metastasized at the time of diagnosis (>50%) (48). The related syndrome, also known as Werner–Morrison syndrome, or watery diarrhea, hypokalemia, and achlorhydria (WDHA) syndrome, is caused by VIP hypersecretion. The cardinal symptom is profuse watery diarrhea typically exceeding 700 mL/day despite fasting (52), reaching up to over 3,000 mL/day in 70% of patients (50), leading to substantial dehydration and hypokalemia. Water and electrolyte loss manifest with lethargy, nausea, vomiting, muscle weakness, cramps, tetany, cardiac arrhythmias, and ultimately hypovolemic shock. Flushing occurs in 80% of patients due to the VIP-induced vasodilation (50).

#### Diagnosis

The diagnosis of VIPoma is made in patients with suggestive symptoms and a VIP serum level three times above the upper limit of normal (ULN) > 60 nmol/L. VIP is characterized by episodic secretion, so repeat measurements are required and, importantly, when the patient is symptomatic. Patients with high circulating VIP levels (≥5 ULN) have a poorer prognosis than those with moderately elevated levels (53). Hypokalemia and hyperchloremic metabolic acidosis occur due to gastrointestinal fluid loss and bicarbonate wasting, and hypochlorhydria due to the direct inhibitory effect of VIP on gastric acid production. Hypercalcemia is caused by dehydration and/or stimulation by VIP of bone resorption. CT/MRI and EUS can easily detect VIPoma, as the primary tumor is usually of a diameter >3 cm. Additional SRI imaging is applied to detect metastatic spread (24). In patients with metastatic VIPoma, CT/MRI is also important in the localization of the disease extension (49).

### Somatostatinoma

#### History and clinical presentation

Somatostatinomas represent less than 1% of functioning PanNENs (54) with an estimated annual incidence of less than 0.1 per million individuals (48). The mean age at diagnosis is 50–55 years with a roughly equal sex distribution (55). Approximately 55% of somatostatinomas are located in the pancreas, and, of these, two-thirds arise within the head of the pancreas. The remainder arises in the ampulla and periampullary region of the duodenum or rarely in the jejunum (56). Notably, more than 60% of somatostatinomas are malignant, and 70–92% present with metastatic disease (57). Their incidence in MEN1 is <1% (3). Hereditary pancreatic somatostatinoma may be more prevalent than previously considered in the setting of von Hippel–Lindau syndrome (VHL) or neurofibromatosis I (NF-1) (57). The somatostatinoma syndrome is caused by somatostatin hypersecretion and is found in <5% with the majority of NENs with immunohistochemical expression of somatostatin being silent (55). Diabetes mellitus (60%), diarrhea/steatorrhea, gallstone disease (70%), and weight loss are associated manifestations due to the inhibitory effects of somatostatin on insulin, cholecystokinin, and pancreatic exocrine enzyme secretion (58). Jaundice and abdominal tenderness may also present in functioning as in non-functioning neoplasms.

#### Diagnosis

The diagnosis is established in patients with suspicious symptoms with an elevated fasting serum somatostatin level of ≥100 pg/mL (54). It has to be noted that according to recent guidelines, neoplasms with somatostatin-positive expression, known as somatostatin-producing well-differentiated NEN, have to be differentiated from somatostatinomas that have to be accompanied by the somatostatinoma syndrome (2, 55). Morphological (3-phase CT, MRI, EUS) and functional imaging modalities are important to localize somatostatinomas in the pancreas or duodenum. Additional SRI imaging has the highest sensitivity for detecting metastases (2, 24).

### Ectopic hormonal syndromes secondary to PanNENs (ACTHoma, PTHrPoma, PanNEN associated with CaS, calcitoninoma and GHRHoma)

Occasionally, 3–6% of PanNENs can also secrete hormones or amines that normally originate from other tissues and can cause distinct clinical syndromes characterized as paraneoplastic syndromes (PNS) (59, 60, 61).

#### History, clinical presentation, and diagnosis

##### ACTHoma

Adrenocorticotropic hormone-producing PanNENs (ACTHomas) are an extremely rare type of PanNEN that often causes ectopic ACTH syndrome (EAS) (62). These neoplasms may be aggressive and difficult to treat. According to a review including 336 patients, ACTHoma was more common in women, with a mean tumor size of 4.3 cm, mostly located in the pancreatic tail (63). Clinically, it manifests with hypokalemia, diabetes, muscular weakness, hypertension, moon face, and edema (62). Diagnosis is based on clinical findings and biochemical hypercortisolism (non-suppressed cortisol after 1 mg overnight dexamethasone suppression, increased 24 h urine free cortisol, increased midnight salivary cortisol) along with increased ACTH levels and finally confirmed by a lack of response in bilateral inferior petrosal sinus sampling (62).

##### PTHrPoma

Parathyroid hormone-related peptide-secreting PanNENs (PTHrPomas) are reported in a few published case reports (64, 65). There appears to be a female preponderance of 4:1 with a mean age of 38.6 years at diagnosis. The clinical syndrome includes symptomatic hypercalcemia presenting with abdominal pain, nausea, vomiting, constipation, and confusion. Biochemical diagnosis is made by the determination of hypercalcemia and hypophosphatemia with high PTHrP levels and suppressed PTH levels (2, 65).

##### PanNEN associated with CaS

PanNENs associated with CaS is a clinical condition caused predominantly by serotonin hypersecretion, has also been called pancreatic carcinoid, or serotonin-secreting PanNEN or serotoninoma, or serotonin-producing enterochromaffin-cell tumor of the pancreas (66, 67). The systemic features of CaS develop when serotonin circumvents the portal and pulmonary arterial circulation and escapes liver and lung metabolism. Diarrhea, flushing, wheezing, and tachycardia are suggestive symptoms. The latter along with elevated 5-hydroxyindoleacetic acid (5HIAA) > 50 μmol/24 h, the main metabolite of serotonin, establishes the diagnosis (67).

##### Calcitoninomas

Calcitonin-secreting PanNENs (calcitoninomas) are also mainly reported as case reports in literature. According to a previous systematic analysis of published cases, most patients presented with metastatic spread at the time of diagnosis. Main symptoms were watery diarrhea and flushing due to calcitonin hypersecretion. Mean serum calcitonin was elevated to 89.2-fold the ULN (68).

##### GHRH-secreting PanNENs

PanNENs which secrete GHRH have been described both as sporadic and in the setting of MEN1. According to a previously published series, 19/25 (76%) of patients with a PanNEN-secreting GHRH displayed MEN1 mutations (69). Twenty-six patients with acromegaly from PanNEN in the context of MEN1 have been described so far (70). Multihormonal secretion may occur in the same individual from either a solitary or several different pancreatic neoplasms. In addition to typical symptoms of acromegaly, patients also develop manifestations from co-secreted hormones (70). Diagnosis of ectopic acromegaly can be challenging, but early diagnosis is important to avoid unnecessary pituitary surgery and disease progression. The most common primary neoplasmatic sites include the lung (53%) and the pancreas (34%). The vast majority of GHRH-secreting neoplasms are expected to be visible on cross-sectional imaging (median diameter 55 mm) (69). Levels of IGF1 > 2xULN and GHRH >250 mg/L confirm diagnosis (2). CT, MRI, and EUS are the imaging modalities used to localize these rare functioning PanNENs. Additional functional imaging (SRI) is performed to detect small lesions and metastatic spread, but the negative findings of ^11^C-methionine-PET increase the suspicion of an ectopic syndrome (71) (Table 1).

**Table 1:** Functioning pancreatic NENs: clinical manifestations, diagnosis, and management.

Functioning pancreatic NENs	Clinical manifestations	Diagnosis	Treatment
Insulinoma	Whipple triad, neuroglycopenic symptoms, weight gain	Biochemical – 72 h fast; imaging (CT/MRI, EUS, SRI: DOTATATE/DOTATOC), ASVS	Surgical resection, dietary modification, diazoxide, SSA, PRRT, local treatments, MTT, chemo
Gastrinoma	Zollinger–Ellison syndrome	Biochemical: gastrin levels; OGD; imaging (CT/MRI, EUS, SRI: DOTATATE/DOTATOC)	Surgical resection, high-dose PPI, SSA, PRRT, locoregional treatments, MTT, chemo
Glucagonoma	Weight loss, necrolytic migratory erythema, diabetes, diarrhea, nutritional deficiencies, DVT	Biochemical: glucagonoma levels; imaging (CT/MRI, EUS, SRI: DOTATATE/DOTATOC)	Surgical resection, amino acid infusion and zinc supplementation, SSA, PRRT, local treatments, MTT, chemo
VIPoma	WDHA syndrome	Biochemical: VIP levels; imaging (CT/MRI, EUS, SRI: DOTATATE/DOTATOC)	Surgical resection, hydration, SSA, glucocorticoids, PRRT, local treatments, MTT, chemo
Somatostatinoma	Diabetes, diarrhea, gallstone disease, weight loss	Biochemical: somatostatin levels; imaging (CT/MRI, EUS, SRI: DOTATATE/DOTATOC)	Surgical resection, SSA, PRRT, local treatments, MTT, chemo
Ectopic hormonal syndromes in pancreatic NENs	Related to ectopic hormone hypersecretion	Biochemical; imaging (CT/MRI, EUS, SRI: DOTATATE/DOTATOC)	Surgical resection, PRRT, locoregional treatments, MTT, chemo, ectopic hormone directed treatment
ACTHoma	Cushing syndrome	UFC, ODS, salivary cortisol levels → ACTH levels → LDDST, HDDST, AVP test, IPSS	Steroidogenic enzyme inhibitors (osilodrostat, metyrapone, ketoconazole, mitotane)
PTHrPoma	Symptomatic hypercalcemia	Increased calcium levels, low PTH levels, increased PTHrP levels	Denosumab, zolendronic acid, cinacalcet
Serotoninoma	Carcinoid syndrome (diarrhea and flushing)	Increased 24 h 5-HIAA urine levels	Telotristat, somatostatin analogs
Calcitoninoma	Watery diarrhea, abdominal pain, weight loss	Increased calcitonin levels	Ondansetron
GHRHoma	Acromegaly	Increased IGF-1 levels ± GH suppression by oral glucose tolerance test	Pegvisomant, cabergoline, somatostatin analogs

ACTH, adrenocorticotropic hormone; ASVS, arterial calcium stimulation with hepatic venous sampling; AVP, arginine-vasopressin; CT, computed tomography; DVT, deep vein thrombosis; EUS, endoscopic ultrasound; GH, growth hormone; GHRH, growth hormone releasing hormone; IGF-1, Insulin-like growth factor 1; HDDST, high-dose dexamethasone suppression test; IGF-1, insulin like growth factor; IPSS, inferior petrosal sinus sampling; LDDST, long low-dose dexamethasone suppression test; MRI, magnetic resonance imaging; MTT, molecular targeted treatment; ODS, overnight dexamethasone suppression; OGD, esophagogastroduodenoscopy; PPI, proton pump inhibitors; PRRT, peptide receptor radionuclide therapy; PTHrP, parathyroid hormone related protein; SRI, somatostatin receptor imaging; SSA, somatostatin analogs; UFC, urine free cortisol; VIP, vasoactive intestinal peptide; WDHA, watery diarrhea, hypokalemia, achlorhydria; 5-HIAA, 5-hydroxyindoleacetic acid.

## Functioning PanNENs related to inherited syndromes

PanNENs are inherited in 17% of patients and are present in the context of monogenic familial endocrine syndromes (2, 72). Distinctive features of syndromic cases are young age at presentation (<40 years), multisystem involvement, multiple neoplasms synchronous or metachronous, and characteristic inheritance patterns. The syndromes associated with PanNENs include MEN1, MEN4, NF1, and tuberous sclerosis complex (TSC) (3). Pancreatic NENs also occur in up to 10–17% of patients with VHL, in their majority, though, as non-functioning tumors. Most of these syndromes are inherited in an autosomal dominant manner. Multiple PanNENs are typical in MEN1, typically non-functioning but co-existence of functional PanNENs has been also reported (73). The functioning PanNENs observed within the spectrum of MEN1 include mostly insulinoma (10%) and gastrinoma, whereas glucagonoma, somatostatinoma, and VIPoma are present in a range of 1% (2). On the other hand, 4–8% of insulinomas, 15–30% of gastrinomas, 6–11% of VIPomas, 5–20% of glucagonomas, and 2–7% of somatostatinomas are associated with MEN1 (74). In MEN4, the cases of PanNENs reported were non-functioning (2). Functioning PanNENs are rare in NF1 with few cases of insulinoma and gastrinoma reported (2). PanNENs are an uncommon finding in TSC, but with a frequency higher than in the general population, and insulinoma has been reported as the predominant functioning tumor (2).

## Treatment approach to functioning PanNENs

In functioning PanNENs, if radical resection is feasible, curative surgery is recommended (2). Control of hormonal hypersecretion is essential for symptom relief and consequent safe planning of the surgical procedure and/or administration of systemic treatment in cases of residual/metastatic disease. The management of functioning PanNEN syndromes requires a multidisciplinary approach consisting of medical, surgical, and interventional treatment.

### Surgical approach

For all PanNENs, surgical resection depends on the localization of the tumor (enucleation, distal pancreatectomy with splenectomy, spleen-preserving distal pancreatectomy, central pancreatectomy, pancreaticoduodenectomy, or total pancreatectomy) and represents the only curative treatment for these neoplasms (2, 11).

For insulinomas, parenchyma-sparing pancreatic resection, laparoscopic or open, with the use of intraoperative ultrasound, if the localization is unknown, is the first-line surgical option (2, 11, 75). Formal lymphadenectomy is not routinely required, since the incidence of nodal metastases is low. Enucleation is an option for patients with insulinomas less than 2 cm, located at ≥ 3 mm from the main pancreatic duct. In cases with locoregional extension or spread to lymph nodes or distant metastases, surgical removal of the neoplasm represents the only potentially curative therapy (11, 76). In metastatic disease, cytoreductive surgery can be considered when ≥70–90% of visible disease can be debulked, and the remaining liver function is adequate (77) to control the functional syndrome and improve the efficacy of systemic or locoregional therapies (2, 11).

Gastrinomas are localized in the duodenum (60–70%), pancreas (20–30%), or lymph nodes (about 10–20%); thus, additional peripancreatic lymphadenectomy is mandatory, since nodal dissection reduces the rate of ZES persistence and improves survival (78, 79). Patients with sporadic ZES and negative preoperative imaging also benefit from surgical exploration with curative intent (2). In cases of recurrence, patients have prolonged survival after reoperation, and 25% of them can be cured with repeat surgery (80). In metastatic disease, surgical removal of resectable disease should be considered to reduce tumor burden and allow better symptom control with medical treatment in the majority of the patients (81). Unlike sporadic gastrinomas, MEN1-gastrinomas are usually multiple, localized in the duodenum, with lymph node metastases at the time of diagnosis, although the primary neoplasms might be microgastrinomas of 1–2 mm (33, 82). The indication, timing, and optimal type of surgery are controversial in MEN1-related ZES. The presence of an imageable pancreatic lesion >20 mm in MEN1-ZES, although most likely not representing the functioning lesion, may be a parameter to indicate surgery (2, 83, 84, 85). However, the excellent long-term survival even without surgery in 75% of these patients does not allow for a consensus on surgical treatment (74). On the other hand, in a case series of MEN1-ZES, pancreatoduodenectomy provided cure over 80% more than 10 years after surgery (83). Interestingly, MEN1 patients with a small pancreatic neoplasm, who did not undergo surgery, but solely medical treatment, showed similar 15-year survival rates (89–100%) to the groups with larger and/or more than one neoplasms that were operated compared with the ones not operated because of diffuse liver metastases (52%) (85).

In glucagonomas, supportive therapy with nutritional supplementation, amino acid infusions, zinc replacement, mineral and vitamin replacement, hyperglycemia control, and prophylactic heparin treatment should be considered preoperatively (47). Urgent removal might be important to alleviate symptoms, as post-surgery, symptoms resolve within weeks (86). VIPomas develop liver metastases at diagnosis in more than 50% of cases (48), and metastasectomy can be an option when resection of all visible metastases is feasible and patients have a good performance status and slow progression, or as debulking therapy in patients with uncontrolled functioning syndrome resistant to the conventional antisecretory therapy, with an immediate symptom control rate for the majority of the patients (53, 87). Somatostatinomas in the body or tail of the pancreas are either enucleated or removed by distal pancreatectomy (88). Neoplasms in the head of the pancreas that cannot be enucleated or smaller tumors with lymph node metastases will mandate a Whipple’s pancreaticoduodenectomy (89, 90). Since somatostatinomas have a high malignant potential (48), metastasectomy when technically feasible, is considered to ameliorate symptoms caused by hormonal hypersecretion (89, 90).

### Endoscopic approach

Endoscopic ultrasound-guided radiofrequency ablation (RFA) might be considered as treatment option for patients with small ≤ 2 cm localized insulinomas who are not fit for surgery (91). Despite the high clinical success rate and safety data reported, further clinical evidence is required to incorporate the method into the routine treatment approach for small insulinomas (2, 11). There are limited data for the EUS-RFA performance in pancreatic gastrinomas. Only a few cases have been reported with a success rate of 50%, but care should be taken in duodenal gastrinomas because of the risk of perforation (2, 92).

### Antisecretory medical treatment

In the context of antisecretory medical treatment, it has to be taken into consideration that hormonal hypersecretion is often characteristic of well-differentiated NENs and, due to the clinical syndrome, can lead to an earlier detection of an NEN, and thus to a better prognosis. Inversely, the hormonal secretion has been shown to negatively affect survival and, particularly in cases of metachronous hormonal syndromes, a PanNEN progression is observed, with higher proliferation (93, 94).

In insulinomas, dietary modification with frequent carbohydrate-rich meals or even intravenous glucose infusion might be needed to control hypoglycemic symptoms prior to surgical intervention of localized disease (2). Diazoxide, an insulin release inhibitor acting on the ATP-sensitive potassium channels on insulinoma cells, at doses of 50–600 mg daily, is the first-line medical therapy for advanced insulinoma, with an estimated efficacy of 50% (95). Glucocorticoids are rarely used because of their serious metabolic effects (dyslipidemia, diabetes, obesity, hypertension) and their immunosuppressive effect and the increased risk of sepsis (11). Verapamil, beta-blockers, and diphenylhydantoin use have also been reported in cases of refractory hypoglycemia with inconsistent results (76). Glucagon therapy available in different formulations (intramuscular, subcutaneous, intranasal) can effectively reverse acute hypoglycemia (11). Somatostatin analogs can reduce insulin levels and prevent hypoglycemic episodes in patients with advanced insulinomas with a reported efficacy of 50–60% (96). As SSAs also decrease glucagon secretion, which could paradoxically aggravate hypoglycemia in cases of reduced expression of SSTR2, a trial with short-acting octreotide under close monitoring of glucose levels is advised before long-releasing formulas are used (2, 11). Second-generation SSA, pasireotide, has also shown promising results not only because of its SSTR5 blocking effect but also because of its hyperglycemic action (2, 11). In advanced insulinoma cases with progressive disease and hypoglycemia refractory to SSAs, treatment with everolimus can effectively decrease insulin secretion leading to notable symptomatic improvement (2, 11). Novel molecules as the monoclonal antibody against the insulin receptor RZ358, ersodetug, have been tested for effectively controlling hyperinsulinemia in a phase 2 multicenter study, while its administration in an insulinoma patient led to control of hypoglycemia (97).

In gastrinomas, PPIs are the first-line treatment to control gastric acid hypersecretion. High doses are frequently administered (60 mg/day omeprazole), with patients diagnosed with MEN1-ZES requiring even higher daily doses (80–120 mg/day omeprazole) (2, 98). H2 blockers are also effective but less used than PPIs because of their lower activity and the need for very high doses (98). Monitoring B12 deficiency, hypomagnesemia, and fracture risk is imperative in patients on long-term high dose PPI (99). SSA treatment can also control gastrin hypersecretion and subsequent gastric acid hypersecretion but less effectively than PPIs. Unless an antitumor effect is required, SSAs are second-line antisecretory agents if PPIs fail at baseline (2, 98). In patients with increased surgical risk, medical therapy with high-dose PPIs with or without SSAs can be considered to control gastrinoma symptoms and growth.

Medical treatment in glucagonoma is based on SSAs, applied either preoperatively (2, 11, 100) or in inoperable cases, with good response in the majority of advanced glucagonomas regardless of the antitumor activity (2). SSA regimens (monthly or 2- to 3-week regimens) in cases of inoperable advanced glucagonomas with disseminated liver metastases resulted in remission of symptoms (11). Diarrhea in VIPoma can be debilitating and life-threatening. Resuscitation with intravenous fluids and electrolytes is warranted in severe cases. The medical first-line treatment for VIPomas is SSA treatment for both preoperative stabilization and in advanced disease (2, 101). SSAs are used when curative surgery is not an option as they can effectively control the symptoms caused by excessive VIP levels and prolong progression-free survival (PFS) (2). Notably, SSAs can be administered concomitantly with other treatment modalities (molecular targeted treatment (MTT), liver-directed therapy (LDT), and chemotherapy) (102). Glucocorticoids may be given in combination or alternatively in secretory diarrhea non-responsive to SSAs with good results (101). SSAs are the first-line medical treatment used to provide relief of symptoms especially diarrhea and weight loss in somatostatinoma patients or to control somatostatin secretion and tumor growth in metastatic and inoperable disease (54, 103, 104).

In ACTH-secreting PanNENs, the first-line medical treatment of Cushing’s syndrome is steroidogenic enzyme inhibitors, such as metyrapone, osilodrostat, or ketoconazole (2, 105, 106). Second-line options include SSAs and dopamine-agonists, since positive responses have been previously described, or glucocorticoid receptor antagonists (107, 108). Bilateral adrenalectomy is considered a rescue therapeutic procedure in severe cases of uncontrolled hormonal hypersecretion (105, 109, 110). In PTHrP-hypersecreting PanNENs, supportive therapy to control hypercalcemia should be administered, with oral and intravenous fluids, denosumab, and zoledronic acid (64, 111). Calcitonin and loop diuretics are administered as second-line options for refractory humoral hypercalcemia of malignancy (64). In a retrospective case series of 9 patients with metastatic pancreatic PTHrPoma, SSA treatment in 7 patients resulted in a temporary normalization of serum calcium levels, with a long-term response observed in two patients (up to 35.2 months) (111).

The cornerstone of medical treatment of CaS is SSAs, importantly in the presurgical stage, to prevent carcinoid crisis (8, 112, 113). At present, according to the ENETS guidelines, first-generation SSAs are recommended, injected once every four weeks, with shortening of the injection interval to 3 or even 2 weeks to achieve symptom control. Long-acting release pasireotide may be considered as an off-label treatment in cases where other SSAs fail to control symptoms (8, 112). According to a recent meta-analysis, SSA treatment provided an estimated 67–68% overall reduction in symptoms with no significant differences in the efficacy of the applied drug (8). In patients with refractory CaS, additional therapies can be applied, inhibiting serotonin synthesis (telotristat ethyl) along with treatments reducing tumor burden (112, 114). SSAs are the first-line medical treatment in ectopic acromegaly management (2); however, in some cases, it has been proven less effective, presumably due to the lack of SSTR-2 in tumor tissue (115). In persistent IGF-1 elevation, pegvisomant and dopamine agonists can be added to control IGF-1 in the context of personalized treatment (2, 116, 117).

### Local treatments

Local treatments can be performed in combination with surgical resection, allowing the treatment of isolated metastases and leading to better symptomatic control (2). Liver-directed therapy (RFA, TAE, TACE, and SIRT radioembolization) may be an additional treatment option in cases of uncontrolled functioning syndrome despite optimal medical therapy (2, 118). The increased morbidity risk associated with these techniques necessitates planned sessions 4–8 weeks apart (2, 119, 120).

### PRRT

Peptide receptor radionuclide therapy represents a promising systemic treatment modality for functioning PanNENs. The prerequisite for its use is the presence of SSTRs as confirmed by a positive SSTR imaging (121). In metastatic insulinoma patients, PRRT resulted in relevant disease and symptom control in published case series (76, 122, 123). In a recent study including 26 patients, reduction in hypoglycemia score was observed in 81% of participants after 2 cycles of PRRT ^90^Y-DOTATOC or ^177^Lu-DOTATOC (123). Hormonal hypersecretion crisis as a consequence of PRRT has been reported in the range of 1–9% (122, 124) resulting in severe hypoglycemia and hypokalemia. In a series of 36 patients with metastatic gastrinoma, PRRT with ^90^Y-DOTATOC alone or in combination with ^177^Lu-DOTATATE resulted in clinical response and morphological response rates in 16 and 33% of cases, respectively, with prolonged survival (125). Symptom deterioration due to hormonal release during/after PRRT warrants careful patient monitoring and preventive management strategies. PRRT employed in individual patients or in small series of patients with metastatic glucagonoma resulted in disease control and improvement of skin lesions and increased PFS (2, 11, 122, 126). In VIPoma, PRRT constitutes a second-line treatment with positive results in symptomatic control and PFS, as demonstrated in two case series (2, 102). PRRT was reported to achieve biochemical and/or metabolic response or stable disease in a small series of metastatic ACTHoma (127). Finally, PRRT with radiolabeled SSAs induced long-term responses ranging from 9 to 49 months in six PTHrP-NET patients (111).

### Molecular targeted therapies

Everolimus, an inhibitor of the PI3K/AKT/mTOR pathway, is an effective treatment for functioning PanNENs, exerting both antineoplastic and antisecretory effects. RADIANT-3 included patients with gastrinoma, glucagonoma, VIPoma, insulinoma, or somatostatinoma; as much as 24% of them demonstrated effective disease control (128). Metastatic insulinomas have also shown effective disease control, but even in the absence of tumor control, a fast hyperglycemic response is noted from 48 h to 2 weeks of administration (11, 128). In a retrospective, multicenter case series, 11 out of 12 patients showed disappearance of hypoglycemic symptoms with a median symptom-free period of 6.5 months (129). A rechallenge with everolimus can also be considered on an individual basis since initial decreased efficacy appears to be transient, caused by the desensitization or the downregulation of mediators involved in the mTOR pathway (96).

On the other hand, sunitinib, a multitarget tyrosine kinase inhibitor, approved for the treatment of advanced/metastatic PanNENs, was administered to 4 patients with advanced insulinomas, in addition to 19 with gastrinoma, 5 with glucagonoma, and one with somatostatinoma in the seminal study that resulted in its approval (130). However, sunitinib treatment has been associated with deterioration of hypoglycemia in some case reports, an association that needs more clarification, obviating its use in advanced insulinomas (11, 131). Everolimus and sunitinib are employed as fourth-line treatment options in advanced gastrinomas, and both gained approval in the aforementioned studies (128, 130). Due to its hyperglycemic effect, everolimus should be used with caution in patients with glucagonoma with careful monitoring of glucose levels (2). Information on everolimus and sunitinib administration in VIPomas is scarce, but everolimus displayed an antisecretory effect in less than 10% of the patients in a case series, while sunitinib presented a symptomatic response ranging from 33 to 100% of the cases in two reports (53, 102). Everolimus is used in addition to SSAs to achieve tumor control and improve CaS symptoms, reducing both 5-HIAA and chromogranin A levels and inhibiting disease progression (67). Everolimus and sunitinib administration in somatostatinomas has only been reported in studies of advanced PanNENs, so that no clear conclusions can be drawn (54, 128, 130). Finally, poor treatment outcomes on everolimus have been described in the few ACTHoma cases published (132).

Recently, cabozantinib, an oral small-molecule inhibitor of multiple tyrosine kinases, including VEGF receptors, MET, AXL, and RET, was approved for both advanced extra-pancreatic and PanNENs by the CABINET trial (133).

### Chemotherapy

Systemic chemotherapy is recommended as a third- or fourth-line option for advanced functioning PanNENs. Common regimens include Tem/Cap and STZ-based therapy. In a prospective, multicenter, randomized phase II trial, Tem/Cap achieved a 40% response rate in grade 1–2 PanNENs (134), whereas, in mixed series, functioning neoplasms showed partial response or stable disease in 68% of the cases, better than the non-functioning that showed response in 55% (135). Chemotherapy has also been used in cases of metastatic insulinoma with refractory hypoglycemia after progression on prior treatments (11, 76). In addition, inhibition of the mTOR pathway during STZ therapy may enhance antitumor activity, providing a rationale for combination treatment or pretreatment with everolimus (136). In gastrinomas, Tem/Cap has been given in mixed series (135, 137), but no response was seen in other schemes including STZ (138, 139). In metastatic glucagonomas, STZ/dacarbazine may be used because STZ selectively damages pancreatic islet cells (11, 47, 139). STZ-based regimens, alone or combined with 5-fluorouracil (5-FU), have also shown efficacy in symptom and disease control in advanced or metastatic VIPomas (140).

## Conclusion

The management of patients with a functioning PanNEN syndrome is complex due to its heterogeneous presentation and multisystem involvement secondary to the secretion of bioactive substances. An additional challenge is the requirement to diagnose and control both hormonal hypersecretion and tumor growth that has to follow a step-by-step algorithm (Fig. 1). Surgery is the standard treatment to obtain a cure or in advanced cases to control symptoms and disease progression combined with locoregional techniques. Since SSTRs are expressed in PanNENs, SSAs are the cornerstone of medical treatment exerting antisecretory and antiproliferative action. PRRT treatment provides additional therapeutic benefit in advanced PanNENs. Molecular targeted treatment and chemotherapy constitute the second-line systemic treatment in metastatic disease with promising findings from trials and pending novel findings from clinical trials in progress. Follow-up is imperative for early detection of recurrence or aggravation of symptoms in patients with partly controlled or refractory symptoms of hormonal hypersecretion. Rarely, a metachronous switch of the secretory component may occur, producing a new phenotype, or an additional functioning syndrome may emerge, underlying the necessity of appropriate history taking, evaluation, and monitoring. Recording and publication of cases and planning of multicenter interventional trials could aid in further and better understanding of clinical features, predictors of disease prognosis, treatment response and, consequently, discovery of new druggable targets.

**Figure 1:**
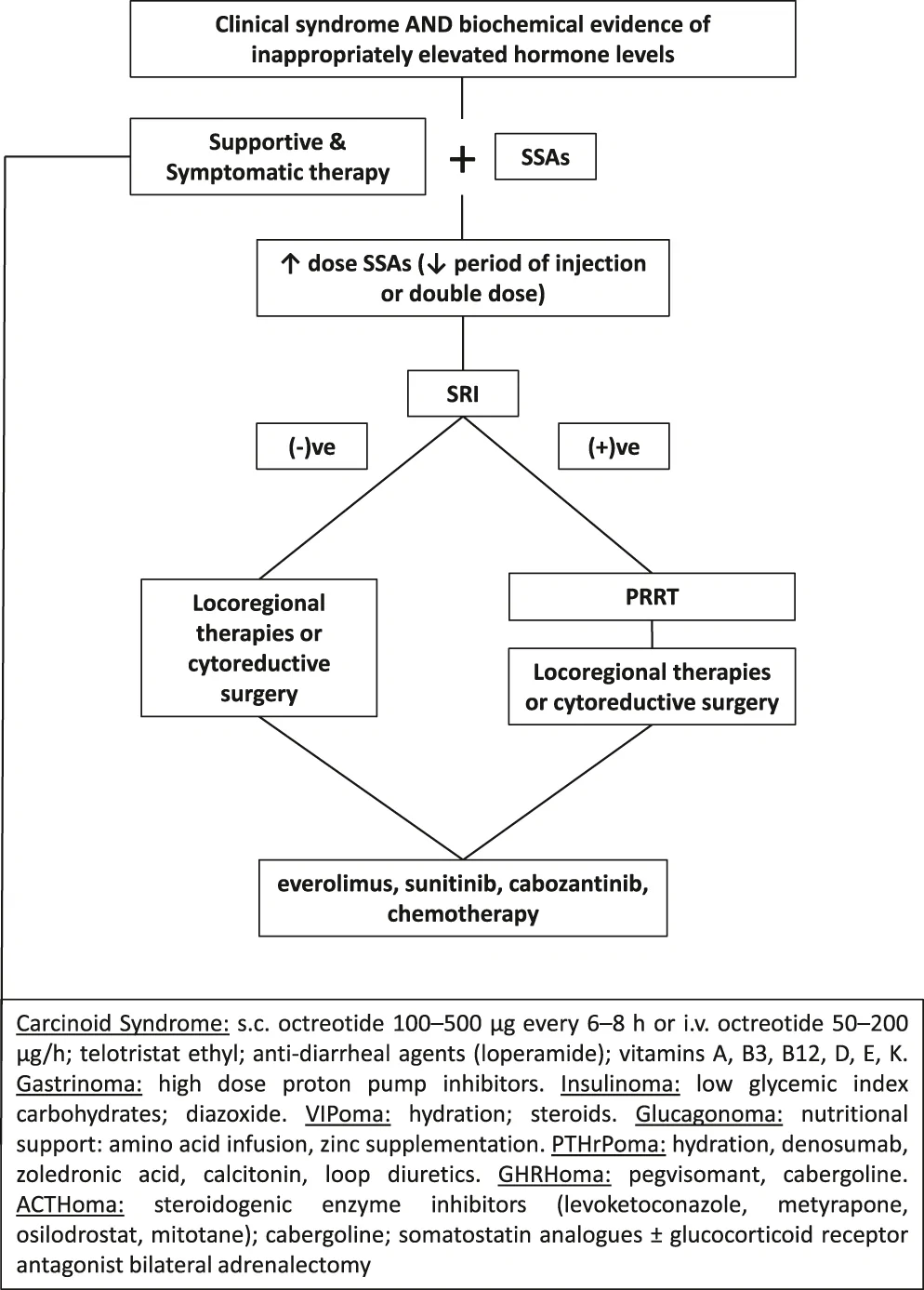
The suspicion of a functional syndrome based on both symptoms and inappropriately elevated hormonal levels dictates a prioritization of hormonal secretion control followed by the control of tumor growth. PRRT, peptide receptor radionuclide therapy; SRI, somatostatin receptor imaging; SSAs, somatostatin analogs; (−)ve, negative; (+), positive; ↑, increased; ↓, decreased.

## Declaration of interest

The authors declare that there is no conflict of interest that could be perceived as prejudicing the impartiality of the work reported.

## Funding

This work did not receive any specific grant from any funding agency in the public, commercial, or not-for-profit sector.
